# Assessing the Efficacy of Mitochondria-Accumulating Self-Assembly Peptides in Pancreatic Cancer: An Animal Study

**DOI:** 10.3390/ijms26020784

**Published:** 2025-01-17

**Authors:** Ho Joong Choi, Seongeon Jin, Junghyun Park, Dosang Lee, Hee Jeong Jeong, Ok-Hee Kim, Ja-Hyoung Ryu, Say-June Kim

**Affiliations:** 1 Department of Surgery, Seoul St. Mary’s Hospital, College of Medicine, The Catholic University of Korea, Seoul 06591, Republic of Korea; hopej0126@catholic.ac.kr (H.J.C.); dosangs@catholic.ac.kr (D.L.); 2 Catholic Central Laboratory of Surgery, College of Medicine, The Catholic University of Korea, Seoul 06591, Republic of Korea; angle49@catholic.ac.kr (J.P.); tps706@naver.com (H.J.J.); ok6201@hanmail.net (O.-H.K.); 3 Department of Chemistry, Ulsan National Institute of Science and Technology (UNIST), Ulsan 44919, Republic of Korea; sungeon3248@unist.ac.kr (S.J.); jhryu@unist.ac.kr (J.-H.R.); 4 Department of Surgery, Eunpyeong St. Mary’s Hospital, College of Medicine, The Catholic University of Korea, Seoul 03312, Republic of Korea; 5 Translational Research Team, Surginex Co., Ltd., Seoul 06591, Republic of Korea

**Keywords:** antioxidant enzymes, mitochondria-targeting moiety, pancreatic cancer, self-assembly peptides

## Abstract

Although pancreatic cancer presents with one of the most unfavorable prognoses, its treatment options are very limited. Mitochondria-targeting moieties, considered a new and prominent treatment modality, are expected to demonstrate synergistic anticancer effects due to their distinct mechanism compared to conventional chemotherapeutic approaches. This study evaluated the therapeutic potential of mitochondria-accumulating self-assembly peptides, referred to as Mito-FFs, utilizing both in vitro and in vivo pancreatic cancer models. Cellular viability assays revealed a concentration-dependent decrease in the survival of MIA-PACA2 pancreatic cancer cells upon exposure to Mito-FF treatment (*p* < 0.05). Subsequent in vitro Mito-FF treatments prompted the use of several molecular analyses, including Real-time PCR, Western blot analysis, and MitoSOX staining, which collectively indicated an upsurge in apoptosis, a concurrent reduction in the antioxidant enzyme expression, and an elevation in mitochondrial ROS levels (*p* < 0.05). In a murine xenograft model of pancreatic cancer, the intravenous administration of Mito-FF yielded a notable reduction in the tumor volume. Moreover, it upregulated the expression of pro-apoptotic markers, such as cleaved PARP and c-caspase 3, while concurrently downregulating the expression of an anti-apoptotic marker, MCL-1, as evidenced by both Western blot analysis and immunohistochemical staining (*p* < 0.05). It also resulted in the reduced expression of antioxidant enzymes like HO-1, catalase, and SOD2 within excised tumor tissues, as confirmed using Western blot analysis (*p* < 0.05). Cumulatively, the findings underscore the significant anticancer efficacy of Mito-FF against pancreatic cancer cells, predominantly mediated through the induction of apoptosis, suppression of antioxidant enzyme expression, and enhancement of mitochondrial ROS levels within the tumor microenvironment.

## 1. Introduction

Mitochondria, known for their diverse functions, have emerged as promising targets in anticancer strategies [1]. The focus of research has shifted towards targeting mitochondrial metabolism, encompassing aspects such as the electron transport chain function, redox signaling pathways, ROS homeostasis, and apoptotic signaling pathways. Notably, mitochondria, despite being integral to the cell, are considered to have originated as independent organisms, separate from the genetic mechanisms of host cells. This unique origin underscores the potential benefits of targeting mitochondria, as it allows for circumventing tumor genetic surveillance mechanisms that often lead to drug resistance [2,3]. Consequently, the delivery of anticancer drugs to mitochondria holds significant clinical relevance, as it could enhance the drug selectivity for cancer cells, overcome drug resistance, and greatly augment the anticancer efficacy [1].

Recent advancements in mitochondrial-targeting therapies for cancer have focused on exploiting the unique characteristics of cancer cell mitochondria, such as their altered membrane potential. A prime example is the triphenylphosphonium (TPP) cation, which has been widely studied for its mitochondrial selectivity due to the electrochemical gradient inherent to these organelles. Beyond TPP, mitochondria-penetrating peptides (MPPs) have garnered attention, characterized by their ability to translocate across biological membranes and localize within the mitochondrial matrix, demonstrating potential as carriers for antitumor agents [4,5]. Furthermore, pyridinium derivatives are being explored for their pro-apoptotic activity through mitochondrial pathways. A notable study reported the accumulation of amyloid (Aβ) peptides in mitochondria through an interaction with the TOMM22 channel, a component of the mitochondrial import machinery, indicating a mechanism for peptide accumulation that may extend to therapeutic applications [6,7]. Moreover, peptides like SS-31 and those containing a mitochondrial-targeting sequence (MTS) conjugated to cell-penetrating peptides offer a broad landscape of mitochondrial modulators with therapeutic potential [8]. These studies underscore the diversity of strategies employed to target and manipulate mitochondrial function, revealing vast potential for the development of novel anticancer therapies.

Mito-FF is a synthetic molecule composed of diphenylalanine (FF), triphenylphosphonium (TPP), and pyrene, designed to selectively target mitochondria [9]. Diphenylalanine enables self-assembly into highly ordered nanostructures through hydrogen bonding and π-π stacking interactions, while TPP, a lipophilic cation, allows for efficient accumulation in the mitochondrial matrix by traversing the inner mitochondrial membrane [10,11]. Pyrene, as a fluorescent probe, facilitates the visualization of Mito-FF’s localization and behavior within cells. The reaction underlying Mito-FF formation is driven by the unique self-assembly properties of diphenylalanine, which aggregates into tubular structures when a critical aggregation concentration (CAC) is reached. Within mitochondria, Mito-FF molecules undergo self-assembly into fibrils that disrupt mitochondrial membranes and functions. This process is thought to increase ROS production and initiate apoptotic pathways, making Mito-FF a promising candidate for overcoming chemotherapy resistance in cancer therapy. The antitumor efficacy of Mito-FF has been demonstrated in gastric and liver cancer animal models [12,13]. In this study, we aimed to investigate whether the promising antitumor effects of Mito-FF could also be observed in pancreatic cancer models, focusing on its mitochondrial-targeting and apoptosis-inducing mechanisms.

## 2. Results

### 2.1. The Effects of Mito-FF on the Viability of MIA-PACA2 Pancreatic Cancer Cells

**Figure 1:**
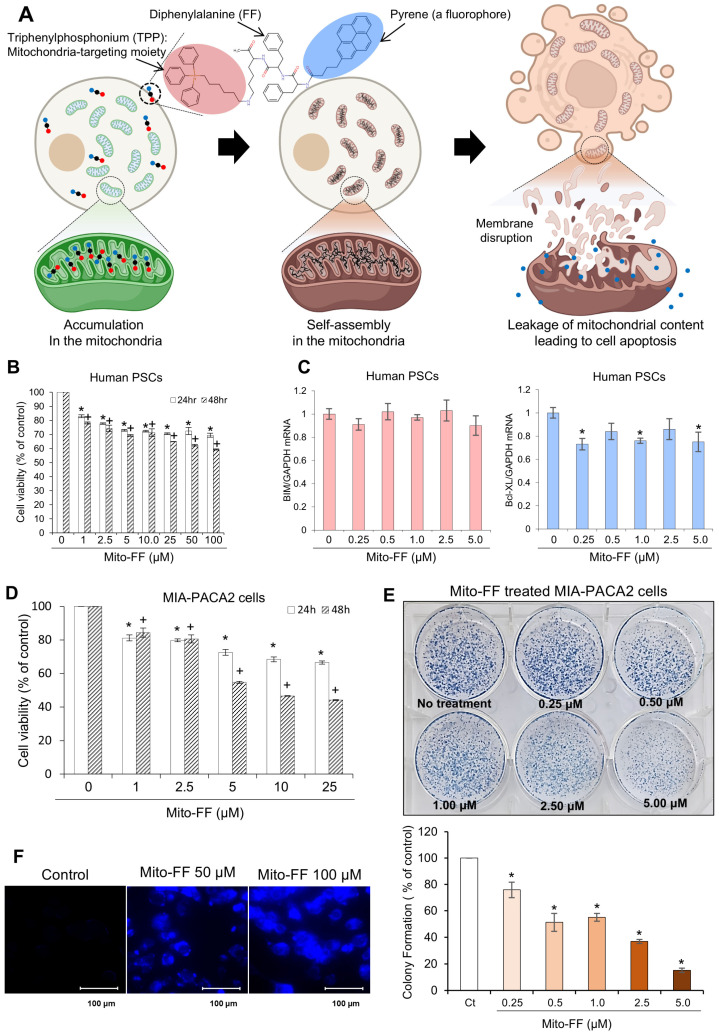
The effects of Mito-FF on MIA-PACA2 pancreatic cancer cells. (**A**) The Mito-FF molecular structure. Mito-FF, a mitochondria-accumulating self-assembly peptide, is composed of three essential components: diphenylalanine, triphenylphosphonium (TPP), and pyrene, which functions as a fluorophore. (**B**) MTT assay results demonstrating the effect of various concentrations of Mito-FF on the viability of human pancreatic stellate cells (PSCs). (**C**) A Real-time PCR analysis depicting the expression levels of pro-apoptotic and anti-apoptotic markers in PSCs treated with Mito-FF. The results indicate no significant changes in the marker expression, suggesting a lack of apoptotic induction by Mito-FF at the concentrations tested. (**D**) The impact of Mito-FF on the cell viability. Cell viability assays demonstrated a concentration-dependent reduction in the MIA-PACA2 pancreatic cancer cell viability following 48 h of Mito-FF treatment (*p* < 0.05). (**E**) The colony assay. Representative images show the effects of Mito-FF (0.25 to 5 μM) on the colony-forming ability of MIA-PACA2 cells after 24 h of treatment. The quantification of the colony formation (% of control) demonstrates a significant concentration-dependent reduction in colonies at all tested concentrations compared to the control (*p* < 0.05). The values are presented as the mean ± the standard deviation of three independent experiments. (**F**) Fluorescence imaging of the Mito-FF uptake in MIA-PACA2 cells. Images showing the intracellular uptake of Mito-FF by tumor cells, with fluorescent tagging facilitating visualization. Control cells (Ctrls) showed minimal fluorescence, while cells treated with 50 µM and 100 µM Mito-FF exhibited an increased fluorescence intensity, indicative of Mito-FF uptake. The values are presented as the mean ± the standard deviation of three independent experiments. Statistical significance is indicated as follows: * denotes *p* < 0.05 compared to the control (untreated) group after 24 h of treatment at each Mito-FF concentration. + denotes *p* < 0.05 compared to the control (untreated) group after 48 h of treatment at each Mito-FF concentration.

Mito-FF is a specific self-assembling peptide designed to selectively target mitochondria. It is composed of three key components: diphenylalanine, triphenylphosphonium (TPP), and pyrene, the latter serving as a fluorophore (Figure 1A). Mito-FFs are anticipated to undergo accumulation within the mitochondrial matrix upon traversing the inner mitochondrial membrane. Within this matrix, these peptides undergo self-assembly, forming fibrous structures. Ultimately, this self-assembly process is expected to result in apoptotic cell death by compromising the integrity of the mitochondria within tumor cells.

In response to the request for the validation of Mito-FF’s side effects on normal cells, we investigated its impact on human pancreatic stellate cells (PSCs) to assess changes in the cell viability and apoptotic factor expression. MTT assays indicated that Mito-FF significantly suppressed the cell viability of human PSCs in a concentration-dependent manner (Figure 1B), as evaluated after 24 h and 48 h treatments. Additionally, Real-time PCR analysis showed no significant concentration-dependent changes in the expression of pro-apoptotic (BIM) or anti-apoptotic (Bcl-xL) markers after 24 h or 48 h treatments (Figure 1C). These results suggest that Mito-FF did not induce apoptotic effects in PSCs at the concentrations tested.

Cell viability assays were employed to investigate the impact of Mito-FF on the MIA-PACA2 pancreatic cancer cell viability. Mito-FF exhibited a concentration-dependent reduction in the viability of MIA-PACA2 pancreatic cancer cells, with a pronounced decrease observed following 48 h of Mito-FF treatment (*p* < 0.05) (Figure 1D). When comparing the cell viability between normal cells (human PSCs) and cancer cells (MIA-PACA2 pancreatic cancer cells), Mito-FF achieved an IC50 at 25 μM in MIA-PACA2 cells, while even at 100 μM, an IC50 was not reached in human PSCs, indicating a stronger inhibitory effect on pancreatic cancer cells than on normal cells. Cell viability assays conducted on additional pancreatic cancer cell lines, including AsPC-1, Capan-1, and Capan-2, confirmed the efficacy of Mito-FF, demonstrating a pattern of response consistent with that observed in the MIA-PACA2 cells (Supplementary Figure S1).

Subsequently, the colony-forming ability of MIA-PACA2 cells was assessed after treatment with various concentrations of Mito-FF (0.25 to 5 μM) for 24 h. The quantification of the colony formation revealed a concentration-dependent reduction in the number of colonies, with significant suppression observed at all tested concentrations compared to the control (*p* < 0.05) (Figure 1E). This result indicates that Mito-FF effectively inhibited the colony-forming potential of MIA-PACA2 cells in a dose-dependent manner. Fluorescence microscopy was utilized to visualize the cellular uptake of Mito-FF, exploiting its inherent fluorescent properties (Figure 1F). Control MIA-PACA2 cells displayed minimal fluorescence, while those treated with 50 µM and 100 µM concentrations of Mito-FFs demonstrated a marked increase in fluorescence. This elevated fluorescence intensity reflected the internalization of Mito-FF, indicating its uptake into the tumor cells.

### 2.2. The Effects of Mito-FF on the Apoptosis of MIA-PACA2 Pancreatic Cancer Cells

**Figure 2:**
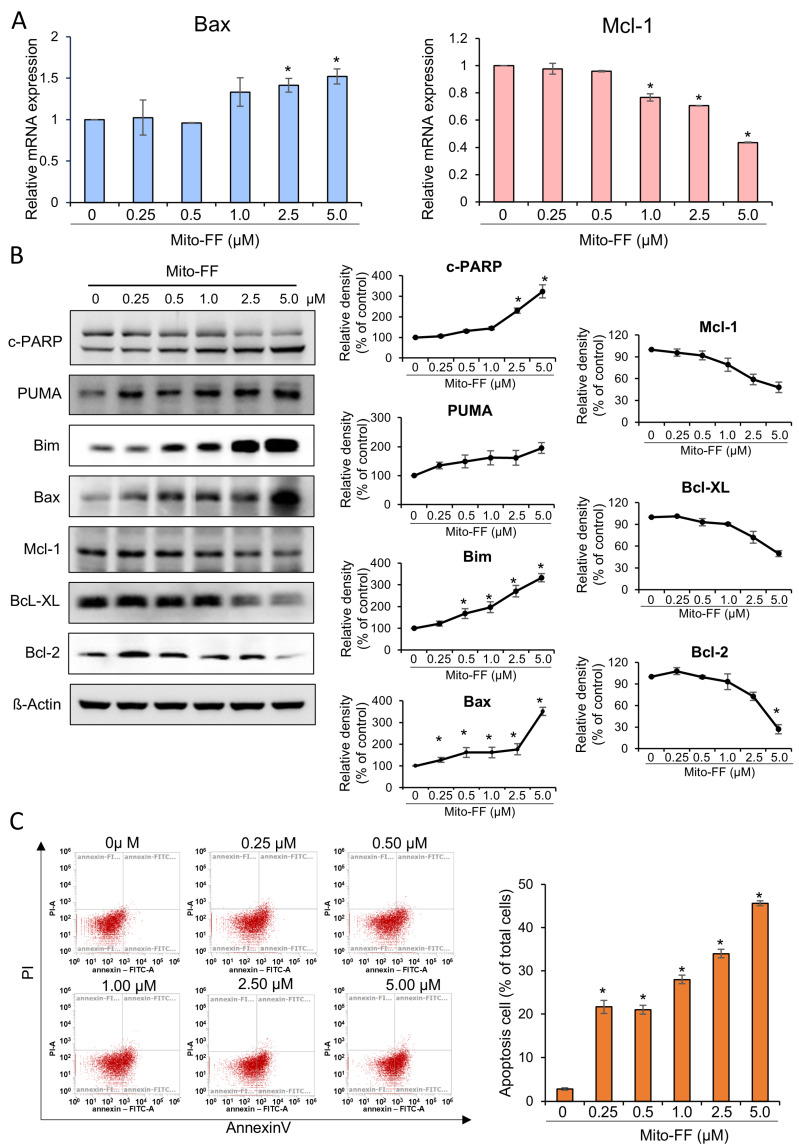
Effects of Mito-FF on apoptosis in MIA-PACA2 pancreatic cancer cells. (**A**) Real-time PCR analysis showing concentration-dependent changes in mRNA expression of apoptosis-related markers Bax and Mcl-1 in MIA-PACA2 cells treated with Mito-FF. (**B**) Western blot analysis demonstrating concentration-dependent alterations in markers related to apoptosis (cleaved PARP, PUMA, Bim, and Bax) and those associated with anti-apoptosis (Mcl-1, Bcl-xL, and Bcl-2) in response to Mito-FF treatment. Relative densities of individual markers were quantified using Image J software (https://imagej.net/ij/download.html, accessed on 14 January 2025). and then were normalized to that of β-actin in each group. (**C**) Effects of Mito-FF on apoptosis of MIA-PACA2 pancreatic cancer cells determined using annexin V/PI staining and flow cytometry. Apoptotic cells were expressed as total percentage of annexin V-positive/PI-negative cells. Number of annexin V-positive cells (early- and late-apoptotic cells) was dose-dependently increased according to increasing concentration of Mito-FF (ranging from 0.25 to 5 μM). Data acquired through flow cytometric analysis were processed and interpreted using Attune NxT Flow Cytometer Software v3.1.2. Values are presented as mean ± standard deviation of three independent experiments. Note: * *p* < 0.05.

Real-time PCR was employed to assess changes in the mRNA expression of the apoptosis-related markers, Bax and Mcl-1, in MIA-PACA2 pancreatic cancer cells following treatment with Mito-FF at varying concentrations. Treatment with Mito-FF demonstrated a concentration-dependent increase in the mRNA expression of the pro-apoptotic marker, Bax, while concurrently decreasing the mRNA expression of the anti-apoptotic marker, Mcl-1 (*p* < 0.05) (Figure 2A). Furthermore, Western blot analysis was conducted to investigate the changes in apoptosis-related markers in response to Mito-FF treatment at different concentrations. Mito-FF exhibited a concentration-dependent augmentation of the expression of pro-apoptotic markers, such as cleaved PARP, PUMA, Bim, and Bax, while simultaneously reducing the expression of the anti-apoptotic markers, such as Mcl-1, Bcl-xL, and Bcl-2 (*p* < 0.05) (Figure 2B).

Next, annexin V/PI staining combined with flow cytometry was used to determine the percentage of cells within a population that were actively undergoing apoptosis. The percentages of apoptotic cells depending on the increasing concentration of Mito-FF were analyzed using flow cytometry. Incubation with an increasing concentration of Mito-FF (0.25–5 μM) for 24 h concentration-dependently increased the percentage of early-apoptotic cells and late-apoptotic cells in MIA-PACA2 pancreatic cancer cells (Figure 2C). Specifically, when treated with a concentration of 5 μM of Mito-FF for 24 h, the percentage of early- and late-apoptotic cells corresponded to 45.7%.

### 2.3. The Effects of Mito-FF on the Oxidative Stress of MIA-PACA2 Pancreatic Cancer Cells

**Figure 3:**
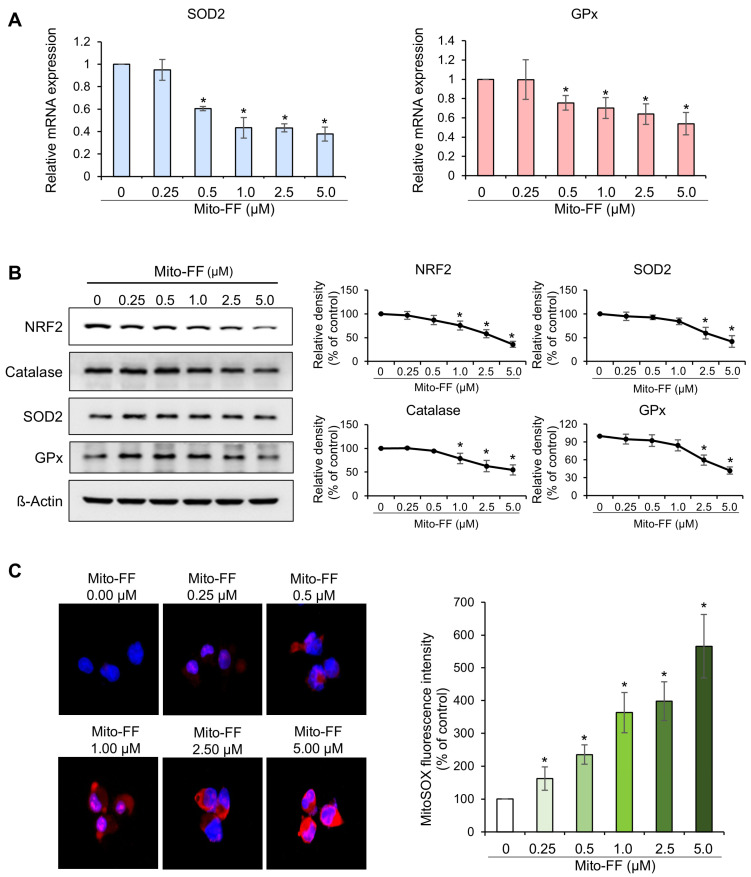
Effects of Mito-FF on oxidative stress in MIA-PACA2 pancreatic cancer cells. (**A**) Real-time PCR analysis of antioxidant enzymes. Treatment of MIA-PACA2 cells with Mito-FF led to concentration-dependent reduction in mRNA expression of antioxidant enzymes, including SOD2 and GPx. (**B**) Western blot analysis and quantification of NRF2, catalase, SOD2, and GPx levels in MIA-PACA2 cells treated with increasing concentrations of Mito-FF (0 to 5 μM). Quantification was performed by normalizing intensity of each band to β-actin, with control (Ct) corresponding to 0 μM Mito-FF treatment. Values represent mean ± standard deviation from three independent experiments. * denotes *p* < 0.05 compared to control group. (**C**) Quantification of MitoSOX red fluorescence. Mito-FF treatment induced concentration-dependent elevation in mitochondrial ROS levels in MIA-PACA2 cells, as evidenced by quantifying MitoSOX red fluorescence. Percentages of immunoreactive areas were measured using NIH image J (https://imagej.net/ij/download.html, accessed on 14 January 2025). and expressed as relative values to those in control tissues. Values are presented as mean ± standard deviation of three independent experiments. Note: * *p* < 0.05.

Real-time PCR was utilized to examine changes in the mRNA expression of antioxidant enzymes, specifically SOD2 and GPx, in MIA-PACA2 pancreatic cancer cells following treatment with varying concentrations of Mito-FF. Treatment with Mito-FF demonstrated a concentration-dependent reduction in the mRNA expression of SOD2 and GPx (*p* < 0.05) (Figure 3A). Subsequently, Western blot analysis was employed to examine alterations in NRF2—a transcription factor that governs the expression of antioxidant enzymes—and in antioxidant enzymes themselves, including catalase, SOD2, and GPx, under varying concentrations of Mito-FF (Figure 3B). In MIA-PACA2 cells treated with increasing concentrations of Mito-FF, a consistent trend of a dose-dependent reduction in antioxidant proteins, including NRF2, catalase, SOD2, and GPx, was observed. Notably, both SOD2 and GPx exhibited an initial transient increase at lower Mito-FF concentrations (0.25–0.5 μM), followed by a marked decrease at higher concentrations (1–5 μM). Despite these initial variations, the overall reduction in antioxidant markers at higher Mito-FF concentrations highlights a significant disruption in the cells’ antioxidant defense system.

The quantification of MitoSOX red fluorescence can serve as an indicator of mitochondrial ROS production, as evidenced by the colocalization of MitoSOX red fluorescence with mitochondrial cytochrome c oxidase. Consequently, to assess the mitochondrial ROS levels following Mito-FF treatment, we quantified the MitoSOX red fluorescence and compared it across varying concentrations of Mito-FF. Notably, Mito-FF exhibited a concentration-dependent elevation in the MitoSOX red fluorescence (*p* < 0.05) (Figure 3C). In summary, it can be inferred that Mito-FF concentration-dependently decreased antioxidant enzymes and increased the mitochondrial ROS levels.

### 2.4. The In Vivo Anticancer Effects of Mito-FF in a Xenograft Model of Pancreatic Cancer

**Figure 4:**
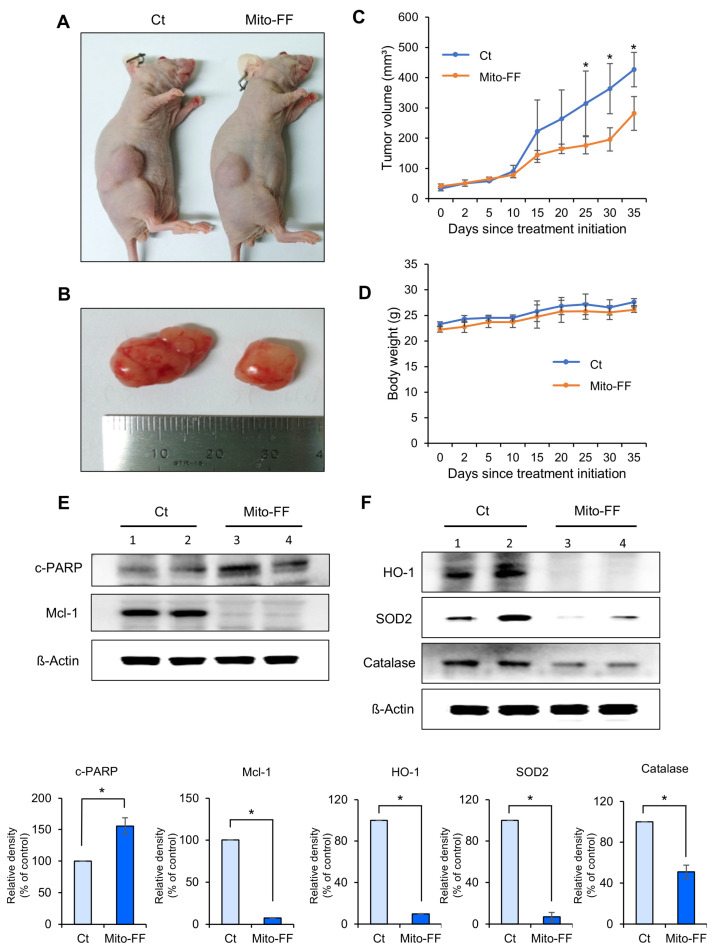
The anticancer effects of Mito-FF in a xenograft model of pancreatic cancer. (**A**) Representative images depicting BALB/c nude mice with established pancreatic cancer xenografts, captured on the 35th day following the initial administration of the experimental treatment. The control group received no Mito-FF treatment, while the Mito-FF group received regular tail vein injections. (**B**) Images of tumors excised from both control and Mito-FF-treated mice, highlighting the difference in the tumor size. (**C**) A graph illustrating the change in the tumor volume over 35 days. The Mito-FF-treated group showed a significant reduction in the tumor size compared to the control group starting from the 25th day (*p* < 0.05). (**D**) A graph comparing the body weight of mice in the control and Mito-FF groups, indicating no significant weight difference between the groups. (**E**) A Western blot analysis of apoptosis markers in the excised tumor tissues. The Mito-FF group showed an increase in the pro-apoptotic marker cleaved PARP and a decrease in the anti-apoptotic marker Mcl-1 compared to the control group (*p* < 0.05). (**F**) A Western blot analysis of antioxidant enzymes in the excised tumor tissues. The Mito-FF group showed a significant reduction in the expression of antioxidant enzymes (HO-1, SOD2, catalase) compared to the control group (*p* < 0.05). The relative densities of individual markers were quantified using Image J software (https://imagej.net/ij/download.html, accessed on 14 January 2025) and then were normalized to that of β-actin in each group. The values are presented as the mean ± the standard deviation of three independent experiments. Note: * *p* < 0.05.

An in vivo xenograft model of pancreatic cancer was established by intramuscularly injecting 1 × 10^6^ MIA-PACA2 pancreatic cancer cells into the buttocks of BALB/c nude mice. This injection was repeated twice per week for a duration of two weeks. Subsequently, Mito-FF was administered via the tail vein in a specified amount twice a week for 35 days. The tumor volume and mouse body weight were measured at 5-day intervals, starting from the initial administration of Mito-FF until the 35th day. On the 35th day, the mice were euthanized, and the tumors were excised for future analysis. Figure 4A,B depict the external appearance of representative xenograft mice from the control and Mito-FF treatment groups, as well as the excised tumors. In a comparison of the tumor volumes, the Mito-FF treatment group exhibited a significant difference compared to the control group, starting from the 30th day of injection (*p* < 0.05) (Figure 4C). In a comparison of the body weights, no significant difference was observed between the control group and the Mito-FF treatment group (Figure 4D).

Western blot analysis was conducted to compare the expression of apoptosis-related markers in excised tumor tissues. In the Mito-FF treatment group, the tumor tissue exhibited a significant increase in the pro-apoptotic marker, cleaved PARP, compared to the control group, while the anti-apoptotic marker, Mcl-1, showed a significant decrease (*p* < 0.05) (Figure 4D). Subsequently, the expression of antioxidant enzymes was also determined using Western blot analysis. In the Mito-FF treatment group, the expression of antioxidant enzymes, including HO-1, SOD2, and catalase, was significantly reduced compared to the control group (*p* < 0.05) (Figure 4E).

### 2.5. The Effects of Mito-FF on the Histological Changes in the Mouse Xenograft Model of Pancreatic Cancer

**Figure 5:**
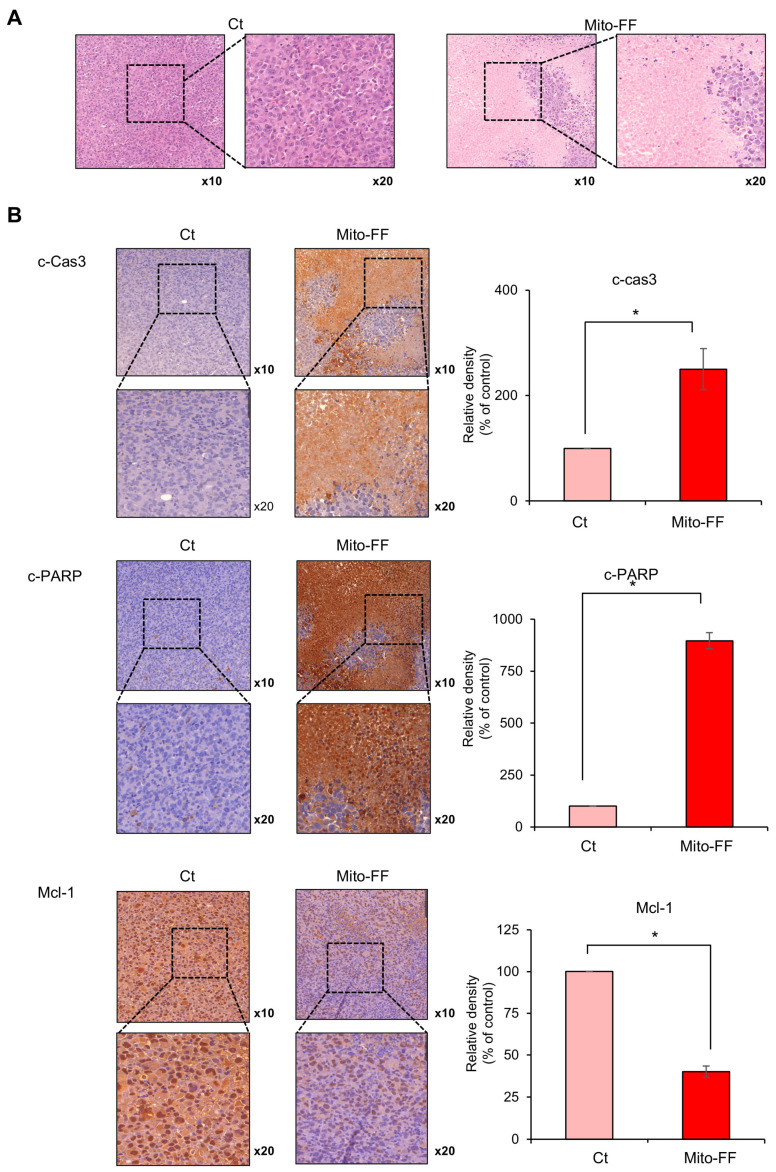
A histological analysis of Mito-FF’s impact on pancreatic cancer xenografts in mice. (**A**) Images showing Hematoxylin and Eosin (H&E)-stained sections of tumor tissues from the xenograft model. The Mito-FF-treated group exhibited a notably reduced tumor cell density compared to the control group. (**B**) An immunohistochemical analysis of apoptotic markers. Staining for c-caspase 3 (**Top**) and cleaved PARP (**Middle**) demonstrated a significant augmentation in the immunoreactive regions within the Mito-FF treatment group, indicative of enhanced apoptotic activity. Conversely, staining for the anti-apoptotic marker Mcl-1 (**Bottom**) revealed a pronounced decrease in immunoreactive areas in the same treatment group, suggesting an amplification of anti-apoptotic processes. The values are presented as the mean ± the standard deviation of three independent experiments. The percentages of immunoreactive areas were measured using NIH image J (https://imagej.net/ij/download.html, accessed on 14 January 2025) and expressed as relative values to those in control tissues. Note: * *p* < 0.05.

Tumor tissues obtained from the mouse xenograft model of pancreatic cancer were subjected to H&E staining for comparative analysis. It was observed that the Mito-FF treatment group exhibited a reduced tumor cell density (Figure 5A). Subsequently, immunohistochemical staining was performed on excised tumor tissues obtained from each group. In an immunohistochemistry analysis of the pro-apoptotic markers, c-caspase 3 and cleaved PARP, the Mito-FF treatment group showed a significantly increased percentage of immunoreactive areas compared to the control group (*p* < 0.05) (Figure 5B, Top and Middle). In an immunohistochemistry analysis of the anti-apoptotic marker, Mcl-1, the Mito-FF treatment group displayed a significantly decreased percentage of immunoreactive areas compared to the control group (*p* < 0.05) (Figure 5B, Bottom).

## 3. Discussion

In this study, the therapeutic efficacy of the novel anticancer compound Mito-FF was assessed in both in vitro and in vivo models of pancreatic cancer. Cell viability assays revealed a concentration-dependent reduction in the viability of MIA-PACA2 pancreatic cancer cells in response to Mito-FF treatment (*p* < 0.05). Following in vitro Mito-FF treatment, several molecular analyses, including Real-time PCR, Western blot analysis, and MitoSOX staining, indicated an increase in apoptosis, a reduction in the expression of antioxidant enzymes, and an elevation in mitochondrial ROS levels. In a mouse xenograft model of pancreatic cancer, the intravenous administration of Mito-FF resulted in a reduced tumor volume. Furthermore, it enhanced the expression of pro-apoptotic markers while reducing the expression of anti-apoptotic markers in excised tumor tissues, as confirmed through various techniques including Real-time PCR, Western blot analysis, and immunohistochemical staining. Collectively, Mito-FF demonstrated significant anticancer effects against pancreatic cancer cells, primarily achieved by promoting apoptosis, diminishing the expression of antioxidant enzymes, and increasing the mitochondrial ROS levels within tumor tissues.

Cancer cells commonly exhibit deviations from “normal” energy metabolism, enabling their survival and proliferation in the challenging microenvironmental conditions frequently encountered within tumors, such as hypoxia and a limited nutrient availability. Mitochondria, serving as the primary source of energy and metabolites within cells, play a pivotal role in this adaptive response. Mitochondrial abnormalities are thus commonly observed in tumor cells, where they undergo metabolic shifts, transitioning from normal oxidative phosphorylation patterns to glycolysis even under aerobic conditions [14]. These alterations in mitochondrial metabolism lead to various adverse outcomes, including elevated levels of ROS, the excessive production of free radicals, the dysregulation of ion channels, and other metabolic changes [3,15]. Consequently, the investigation of strategies aimed at modulating these altered mitochondrial metabolic pathways has emerged as a prominent focus in cancer research. Given the multifaceted roles of mitochondria in energy metabolism, apoptosis regulation, and cell signaling, mitigating the toxic effects on normally metabolizing cells represents an area where mitochondria-targeted agents hold significant therapeutic promise, distinguishing them from other targeted anticancer agents.

Self-assembly represents an equilibrium process involving the transition from individual building units to their aggregated state, necessitating a concentration surpassing the critical aggregation concentration (CAC) [16,17]. However, achieving the CAC within living cells presents challenges due to the complex chemical environment, which hinders interactions among synthetic building units. Intracellular self-assembly thus demands higher molecular concentrations than the CAC, limiting its practical applications. To address this limitation, the concept of Organelle-Localized Induced Supramolecular Self-Assembly (OLISA) was utilized in this study as a general strategy to induce self-assembly within cells by elevating the local concentrations of self-assembling molecules without the need for additional external treatments [9]. This approach relies on small molecules capable of diffusing through the cell membrane reaching specific organelles or subcellular compartments based on their targeting moieties. Within these targeted organelles, the molecules undergo self-assembly due to the increased local concentration, exploiting the fact that the accumulation of molecules inside organelles, such as mitochondria, can be 500–1000 times higher than in the extracellular space [9]. Unlike Enzyme-Instructed Intracellular Self-Assembly (EISA), which typically requires very high concentrations of molecules (several hundreds of micromoles) and often occurs within the cell or near the cell surface [18,19,20], OLISA operates at lower dosages (several tens of micromoles), providing a distinct advantage.

**Figure 6:**
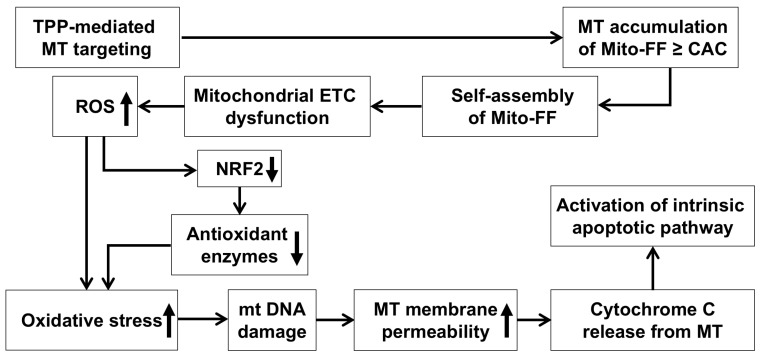
A possible mechanism by which Mito-FF leads to cell apoptosis through oxidative stress. The diagram depicts the proposed mechanism by which Mito-FF induces apoptosis in cancer cells through oxidative stress. The process begins with the mitochondrial targeting of Mito-FF, driven by its TPP moiety and the mitochondrial membrane potential. Once localized in the mitochondria, Mito-FF reaches the critical aggregation concentration (CAC), initiating self-assembly into nanostructures that disrupt the mitochondrial membrane integrity. This disruption impairs the electron transport chain (ETC), leading to excessive ROS production. Elevated ROS levels result in the depletion of antioxidant enzymes and cause oxidative damage to mitochondrial DNA (mtDNA) and proteins, exacerbating the mitochondrial dysfunction. As the mitochondrial damage progresses, the membrane permeability increases, allowing for the release of pro-apoptotic factors like cytochrome c. When cytochrome c is released from mitochondria, it activates the intrinsic apoptotic pathway, leading to caspase activation and apoptosis. Abbreviations: CAC, critical aggregation concentration; ETC, electron transport chain; MT, mitochondria; mtDNA, mitochondrial DNA; TPP, triphenylphosphonium.

A significant advantage of OLISA lies in its ability to activate the intended function upon self-assembly within the specific targeted organelle, a feature effectively demonstrated through the use of the mitochondria-accumulating amphiphilic peptide Mito-FF [12,13]. Mito-FF comprises diphenylalanine as a β-sheet-forming building block, TPP as a mitochondrial-targeting moiety, and pyrene as a fluorescent probe. Mito-FF selectively accumulates in the mitochondria of cancer cells due to their elevated negative membrane potential compared to normal cells [21,22]. This targeting is facilitated by the TPP cation in Mito-FF, which exploits the mitochondrial membrane potential differences to penetrate and accumulate within cancer cells (Figure 6). The rapid division and high metabolic demand of cancer cells further amplify this effect, utilizing the Warburg effect, which lowers the membrane potential significantly. Consequently, Mito-FF is less likely to accumulate in normal cells, where the mitochondrial membrane potential is less conducive to the accumulation of TPP-conjugated molecules. The increased concentration of Mito-FF inside the mitochondria drives its self-assembly into a fibrous structure. Notably, this fibril formation is absent in normal cells. The rigid Mito-FF fibrils disrupt the mitochondrial membrane and activate the intrinsic apoptotic pathway in cancer cells.

The mechanism of action for Mito-FF can be understood through its ability to selectively target mitochondria and disrupt their function, as demonstrated in our study. Mito-FF is believed to accumulate in mitochondria due to its TPP moiety, which takes advantage of the negative mitochondrial membrane potential [23]. Once localized, Mito-FF likely undergoes self-assembly into nanostructures, disrupting the mitochondrial membrane integrity and depolarizing the mitochondrial membrane [24]. This disruption is proposed to impair the electron transport chain (ETC), leading to an overproduction of ROS, including superoxide (O_2_⁻), hydrogen peroxide (H_2_O_2_), and hydroxyl radicals (OH•), as evidenced by the significant reduction in NRF2, SOD2, catalase, and GPx in our study, indicating a depletion of antioxidant defenses [25]. The excessive ROS levels are expected to cause oxidative damage to mitochondrial DNA and proteins, creating a feedback loop that exacerbates mitochondrial dysfunction [26]. This is likely to increase the mitochondrial membrane permeability, as seen from the release of pro-apoptotic factors such as cytochrome c, which then triggers downstream apoptotic cascades. Cytochrome c is hypothesized to interact with Apaf-1, forming the apoptosome, which activates caspase-9 and subsequently caspase-3 and caspase-7, as supported by our data showing an increased expression of cleaved caspases (c-Cas8, c-Cas9, and c-Cas3) [27,28]. Simultaneously, ROS overproduction appears to downregulate anti-apoptotic proteins, such as Bcl-2 and Bcl-XL, while upregulating pro-apoptotic proteins like Bax and Bak, further amplifying the apoptotic signaling pathway [29,30]. This dual mechanism—ROS-mediated mitochondrial dysfunction and the activation of intrinsic apoptotic pathways—highlights the therapeutic potential of Mito-FF in overcoming chemotherapy resistance in pancreatic cancer. These findings are consistent with the observed dose-dependent reduction in antioxidant proteins and the increase in apoptotic markers in our experiments.

## 4. Materials and Methods

### 4.1. Cell Culture

MIA-PACA2, AsPC-1, Capan-1, and Capan-2 pancreatic cancer cell lines were sourced from the Korea Cell Line Bank (KCLB, Seoul, Republic of Korea). These cells were cultured in a DMEM/High (Hyclone; Logan, UT, USA) medium supplemented with 10% fetal bovine serum (Hyclone) and 1% Penicillin–Streptomycin (GibcoBRL; Carlsbad, CA, USA). Human pancreatic stellate cells (HPSCs, iXCells Biotechnologies; San Diego, CA, USA) were also included in the study to evaluate off-target effects and were maintained under identical culture conditions. The cell cultures were maintained at 37 °C in a humidified environment with 5% CO_2_ in an incubator. To verify the authenticity of our cell lines and prevent the potential issue of cell line misidentification, we conducted short tandem repeat (STR) profiling on all cell lines before initiating experiments. This step ensured the validity of our results and supported the reproducibility of our study.

### 4.2. Mito-FF Preparation

Mito-FF was synthesized following standard solid-phase peptide synthesis methods. The peptide backbone, comprising diphenylalanine (FF) and lysine (K), was conjugated with 1-pyrene-carboxylic acid to enhance self-assembly through π-π stacking interactions and hydrophobic effects. The lysine side chain was further conjugated with TPP for efficient mitochondrial targeting, leveraging the mitochondrial membrane potential. The resulting product, Mito-FF, was purified using high-performance liquid chromatography (HPLC) to achieve >99% purity. For experimental use, Mito-FF was dissolved in dimethyl sulfoxide (DMSO) to prepare a 10 mM stock solution. For in vitro assays, the stock solution was diluted with a culture medium to a final DMSO concentration of <0.1%, ensuring there was no solvent-induced cytotoxicity. For in vivo studies, the peptide was further diluted in sterile PBS and administered intravenously to mice at 50 μg/kg body weight twice per week for 35 days. Each solution was freshly prepared before use to maintain the stability and biological activity.

### 4.3. Cell Viability Assay

The cell viability of MIA-PACA2 pancreatic cancer cells was assessed utilizing the Ez-Cytox Cell Viability Assay Kit (Itsbio, Seoul, Republic of Korea) following the guidelines provided by the manufacturer. In all in vitro experiments, DMSO-treated cells served as the vehicle control to ensure that the effects observed were specifically due to Mito-FF and not to the solvent. This control was critical for validating the specificity and efficacy of the treatment.

### 4.4. Colony Formation Assay

A colony-forming assay was conducted using 6-well plates. Cells were seeded at a density of 1000 cells per well in 4 mL of a complete culture medium. Two distinct concentrations of Mito-FF were administered to the cells for a duration of 7 days. Subsequently, the formed colonies were stained with a 0.2% methylene blue solution and quantified.

### 4.5. Real-Time PCR

Total RNA was extracted from both MIA-Paca2 cells using TRIzol reagent (Invitrogen, Carlsbad, CA, USA). Reverse transcription was carried out using an RT premix kit (TOYOBO, Osaka, Japan) with 1 µg of RNA, following the manufacturer’s instructions. For the SYBR Green real-time quantitative polymerase chain reaction (PCR), the following primers were used: human SOD2, forward 5′-GCATCTGTTGGTGTCCAAGG-3′ and reverse 5′-CTGTTGTTCCTTGCAGTGG-3′; human GPx, forward 5′-TCGAGAAGTGCGAGGTGAAC-3′ and reverse 5′-AGCTTGGGGTCGGTCATAAG-3′; human Mcl-1, forward 5′-GGGCAGGATTGTGACTCTCATT-3′ and reverse 5′-GATGCAGCTTTCTTGGTTTATGG-3′; human Bax, forward 5′-TGGAGCTGCAGAGGATGATTG-3′ and reverse 5′-GAAGTTGCCGTCAGAAAACATG-3′; and human GAPDH, forward 5′-GCACCGTCAAGGCTGAGAAC-3′ and reverse 5′-TGGTGAAGACGCCAGTGGA-3′. PCR reactions were conducted using the Applied Biosystems StepOnePlus Real-time PCR system (Thermo, Carlsbad, CA, USA).

### 4.6. Western Blot Analysis

MIA-PACA2 pancreatic cancer cells and mouse tissues were lysed using the EzRIPA Lysis kit (ATTO Corporation; Tokyo, Japan) and quantified using the Bradford reagent (Bio-Rad, Hercules, CA, USA). Western analysis was performed with primary antibodies (dilution of 1:1000) from Cell Signaling Technology (Beverly, MA, USA), followed by HRP-conjugated secondary antibodies (dilution 1:2000) from Vector Laboratories (Burlingame, CA, USA). Specific immune complexes were visualized using the Western Blotting Plus Chemiluminescence Reagent (Millipore, Bedford, MA, USA). Primary antibodies against NRF2, catalase, SOD2, GPx, cleaved PARP, MCL-1, PUMA, HO-1, and β-actin, as well as horseradish peroxidase (HRP)-conjugated secondary antibodies, were sourced from Cell Signaling Technology (Beverly, MA, USA).

### 4.7. Quantification of Apoptosis Using Flow Cytometry

Apoptosis detection involved staining cells with annexin V/PI, utilizing the FITC Annexin V apoptosis detection kit from BD Biosciences (Flanklin Lakes, NJ, USA). Following a 15 min incubation in darkness at 25 °C, a flow cytometric analysis (Thermo) was performed to analyze the cells.

### 4.8. MitoSOX Red Staining

MIA-PACA2 pancreatic cancer cells were grown on Lab-Tek chamber slides (Thermo Fisher Scientific; Waltham, MA, USA). Following a 24 h treatment with Mito-FF, the cells were subjected to staining with 10 µM MitoSOX reagent (Thermo Fisher Scientific) at 37 °C for 10 min. Subsequently, the mitochondrial ROS levels were visualized using the M5000 fluorescence imaging system (EVOS, Invitrogen, CA, USA).

### 4.9. Animals and Study Design

Five-week-old male BALB/c nude mice (Orient Bio, Seongnam, Republic of Korea) were utilized to establish a comparative model of subcutaneous tumor growth. MIA-PACA2 pancreatic cancer cells (5 × 10^6^ cells) were subcutaneously inoculated into each mouse. All animal procedures were conducted in accordance with the guidelines of the Institute for Laboratory Animal Research, the Catholic University of Korea (IRB No: CUMC-2020-0113-03). Two weeks following the injection of tumor cells, all mice developed measurable tumors. To evaluate the in vivo efficacy, the mice were randomly divided into groups (n = 5 per group) and subjected to intravenous treatment with a control and Mito-FF (50 µg/mL, equivalent to 11.80 μM, in 100 µL PBS, administered twice a week) for 35 days. The tumor dimensions were measured biweekly using a caliper, and the tumor volume (V) was determined using the formula length × width^2^ × 0.5236. Upon the completion of the treatment period, all mice were euthanized.

### 4.10. Immunohistochemical Analysis

For the immunohistochemical analysis, tissue sections that had been formalin-fixed and paraffin-embedded underwent deparaffinization and rehydration through an ethanol series, followed by epitope retrieval using standard protocols. Antibodies specific to cleaved caspase 3, cleaved PARP, and MCL-1 were employed for immunofluorescence staining (all antibodies were sourced from Cell Signaling Technology). Subsequently, the samples were examined under a laser scanning microscope (Eclipse TE300; Nikon, Tokyo, Japan) to assess the expression of these antibodies.

### 4.11. Statistical Analysis

All data were analyzed utilizing SPSS 11.0 software (SPSS Inc., Chicago, IL, USA) and were presented as the mean ± the standard deviation (SD). Statistical comparisons among groups were performed using the Kruskal–Wallis test. Probability values of *p* < 0.05 were considered statistically significant.

## 5. Conclusions

Targeting mitochondria and subsequently inducing their disruption through self-assembly peptides represents a novel approach in anticancer therapeutics, effectively circumventing issues related to chemotherapy resistance. This study substantiated the therapeutic efficacy of the novel mitochondrial-targeting peptide Mito-FF in both in vitro and in vivo models of pancreatic cancer. In both models of pancreatic cancer, Mito-FF treatment consistently exhibited potent anticancer effects by elevating apoptosis, reducing the antioxidant enzyme expression, and enhancing mitochondrial ROS production. Therefore, it emerges that this strategy of targeting mitochondria and subsequently inducing their disruption using self-assembly peptides may represent an effective approach for the treatment of pancreatic cancer. Further investigations are warranted to validate these findings.

## Data Availability

The original contributions presented in this study are included in the article/Supplementary Material. Further inquiries can be directed to the corresponding author(s).

## Supplementary Materials

The supporting information can be downloaded at: https://www.mdpi.com/article/10.3390/ijms26020784/s1.

## References

[1] Dong L., Gopalan V., Holland O., Neuzil J. (2020). Mitocans Revisited: Mitochondrial Targeting as Efficient Anti-Cancer Therapy. Int. J. Mol. Sci..

[2] Macasoi I., Mioc A., Mioc M., Racoviceanu R., Soica I., Cheveresan A., Dehelean C., Dumitrascu V. (2020). Targeting Mitochondria through the Use of Mitocans as Emerging Anticancer Agents. Curr. Med. Chem..

[3] Guerra F., Arbini A.A., Moro L. (2017). Mitochondria and cancer chemoresistance. Biochim. Biophys. Acta Bioenerg..

[4] Cheng X., Feng D., Lv J., Cui X., Wang Y., Wang Q., Zhang L. (2023). Application Prospects of Triphenylphosphine-Based Mitochondria-Targeted Cancer Therapy. Cancers.

[5] Pawar A., Korake S., Pawar A., Kamble R. (2023). Delocalized Lipophilic Cation Triphenyl Phosphonium: Promising Molecule for Mitochondria Targeting. Curr. Drug Deliv..

[6] Mayer A., Nargang F.E., Neupert W., Lill R. (1995). MOM22 is a receptor for mitochondrial targeting sequences and cooperates with MOM19. EMBO J..

[7] Wilkins H.M. (2023). Interactions between amyloid, amyloid precursor protein, and mitochondria. Biochem. Soc. Trans..

[8] Xiao Q., Dong X., Yang F., Zhou S., Xiang M., Lou L., Yao S.Q., Gao L. (2021). Engineered Cell-Penetrating Peptides for Mitochondrion-Targeted Drug Delivery in Cancer Therapy. Chemistry.

[9] Jeena M.T., Palanikumar L., Go E.M., Kim I., Kang M.G., Lee S., Park S., Choi H., Kim C., Jin S.M. (2017). Mitochondria localization induced self-assembly of peptide amphiphiles for cellular dysfunction. Nat. Commun..

[10] Yan X., Zhu P., Li J. (2010). Self-assembly and application of diphenylalanine-based nanostructures. Chem. Soc. Rev..

[11] Frantz M.C., Wipf P. (2010). Mitochondria as a target in treatment. Environ. Mol. Mutagen..

[12] Kim D.J., Jeena M.T., Kim O.H., Hong H.E., Seo H., Ryu J.H., Kim S.J. (2020). Novel Therapeutic Application of Self-Assembly Peptides Targeting the Mitochondria in In Vitro and In Vivo Experimental Models of Gastric Cancer. Int. J. Mol. Sci..

[13] Hong T.H., Jeena M.T., Kim O.H., Kim K.H., Choi H.J., Lee K.H., Hong H.E., Ryu J.H., Kim S.J. (2021). Application of self-assembly peptides targeting the mitochondria as a novel treatment for sorafenib-resistant hepatocellular carcinoma cells. Sci. Rep..

[14] Yang Y., An Y., Ren M., Wang H., Bai J., Du W., Kong D. (2023). The mechanisms of action of mitochondrial targeting agents in cancer: Inhibiting oxidative phosphorylation and inducing apoptosis. Front. Pharmacol..

[15] Awais N., Satnarine T., Ahmed A., Haq A., Patel D., Hernandez G.N., Seffah K.D., Zaman M.A., Khan S. (2023). A Systematic Review of Chemotherapeutic Regimens Used in Pancreatic Cancer. Cureus.

[16] Novo M., Freire S., Al-Soufi W. (2018). Critical aggregation concentration for the formation of early Amyloid-beta (1-42) oligomers. Sci. Rep..

[17] Lyubchenko Y.L. (2023). Protein Self-Assembly at the Liquid-Surface Interface. Surface-Mediated Aggregation Catalysis. J. Phys. Chem. B.

[18] Zhang H., Wang Z., Gao T., Wang Z., Ren C., Liu J. (2023). An enzyme-instructed self-assembly system induces tumor acidosis via sequential-dual effect for cancer selective therapy. Acta Biomater..

[19] Shum J., Lee L.C., Chiang M.W., Lam Y.W., Lo K.K. (2023). A Concerted Enzymatic and Bioorthogonal Approach for Extra- and Intracellular Activation of Environment-Sensitive Ruthenium(II)-Based Imaging Probes and Photosensitizers. Angew. Chem. Int. Ed. Engl..

[20] Liu Z., Guo J., Qiao Y., Xu B. (2023). Enzyme-Instructed Intracellular Peptide Assemblies. Acc. Chem. Res..

[21] Fulda S., Galluzzi L., Kroemer G. (2010). Targeting mitochondria for cancer therapy. Nat. Rev. Drug Discov..

[22] Horton K.L., Pereira M.P., Stewart K.M., Fonseca S.B., Kelley S.O. (2012). Tuning the activity of mitochondria-penetrating peptides for delivery or disruption. Chembiochem.

[23] Murphy M.P. (2009). How mitochondria produce reactive oxygen species. Biochem. J..

[24] Fulda S., Kroemer G. (2011). Mitochondria as therapeutic targets for the treatment of malignant disease. Antioxid. Redox Signal..

[25] Kowaltowski A.J., Vercesi A.E. (1999). Mitochondrial damage induced by conditions of oxidative stress. Free Radic. Biol. Med..

[26] Cai J., Yang J., Jones D.P. (1998). Mitochondrial control of apoptosis: The role of cytochrome c. Biochim. Biophys. Acta.

[27] Galluzzi L., Vitale I., Aaronson S.A., Abrams J.M., Adam D., Agostinis P., Alnemri E.S., Altucci L., Amelio I., Andrews D.W. (2018). Molecular mechanisms of cell death: Recommendations of the Nomenclature Committee on Cell Death 2018. Cell Death Differ..

[28] Kroemer G., Zamzami N., Susin S.A. (1997). Mitochondrial control of apoptosis. Immunol. Today.

[29] Green D.R., Kroemer G. (2004). The pathophysiology of mitochondrial cell death. Science.

[30] Youle R.J., Strasser A. (2008). The BCL-2 protein family: Opposing activities that mediate cell death. Nat. Rev. Mol. Cell Biol..

