# Supplementary material for: Assessing the Efficacy of Mitochondria-Accumulating Self-Assembly Peptides in Pancreatic Cancer: An Animal Study
Source: Int J Mol Sci. 2025 Jan 17;26(2):784. doi: 10.3390/ijms26020784 (PMC11766353; doi:10.3390/ijms26020784)
Supplement: Supplementary file 1 [file ijms-26-00784-s001.zip › ijms-3327845-supplementary.pdf]

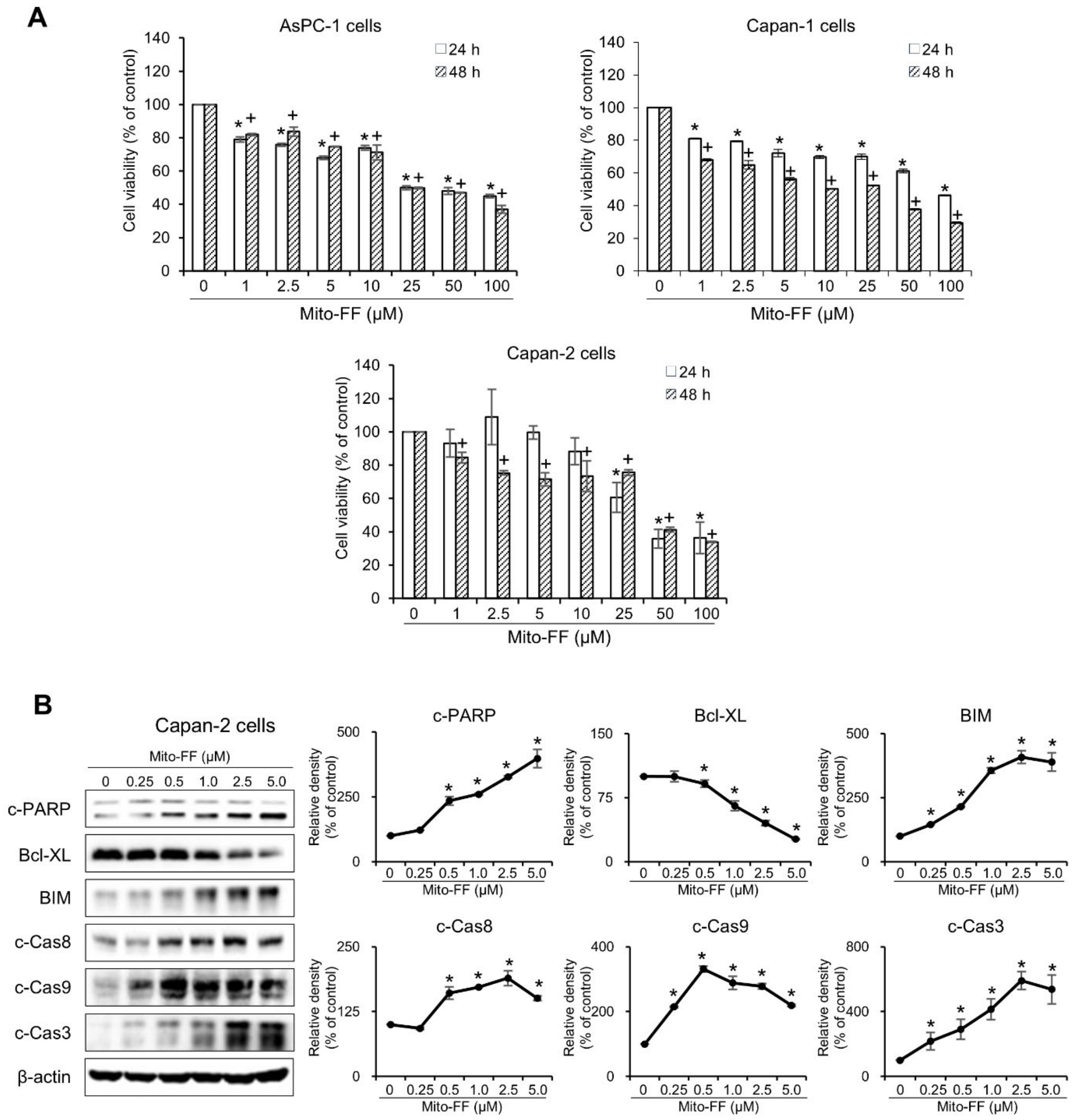

Supplementary Figure S1. Analysis of Cell Viability and Apoptotic Markers in Pancreatic Cancer Cells.

(A) Cell viability assays for ASPC-1 (top), CAPAN-1 (middle), and CAPAN-2 (bottom) pancreatic cancer cells, depicting the concentration-dependent effects of Mito-FF. Statistical significance is indicated as follows: * denotes $P < 0.05$ compared to the Mito-FF 0 μM group after 24-hour treatment, while + denotes $P < 0.05$ compared to the Mito-FF 0 μM group after

48-hour treatment.

(B) Western blot analysis displaying the expression of apoptosis-related markers (c-PARP, Bcl-XL, BIM, c-Cas8, c-Cas9, and c-Cas3) in CAPAN-2 cells, demonstrating the apoptotic response to various concentrations of Mito-FF. Quantification of relative band intensities was normalized to β-Actin. Statistical significance (*) indicates $P < 0.05$ compared to the Mito-FF 0 μM group.
